# Optimization‐based framework for resin selection strategies in biopharmaceutical purification process development

**DOI:** 10.1002/btpr.2479

**Published:** 2017-05-18

**Authors:** Songsong Liu, Spyridon Gerontas, David Gruber, Richard Turner, Nigel J. Titchener‐Hooker, Lazaros G. Papageorgiou

**Affiliations:** ^1^ Centre for Process Systems Engineering, Dept. of Chemical Engineering University College London Torrington Place London WC1E 7JE UK; ^2^ EPSRC Centre for Innovative Manufacturing in Emergent Macromolecular Therapies, University College London Gordon Street London WC1H 0AH UK; ^3^ School of Management Swansea University Bay Campus, Fabian Way Swansea SA1 8EN UK; ^4^ MedImmune Limited, Milstein Building Granta Park Cambridge CB1 6GH UK

**Keywords:** resin selection, purification process development, multiobjective optimization, ε‐constraint method, Dinkelbach's algorithm

## Abstract

This work addresses rapid resin selection for integrated chromatographic separations when conducted as part of a high‐throughput screening exercise during the early stages of purification process development. An optimization‐based decision support framework is proposed to process the data generated from microscale experiments to identify the best resins to maximize key performance metrics for a biopharmaceutical manufacturing process, such as yield and purity. A multiobjective mixed integer nonlinear programming model is developed and solved using the ε‐constraint method. Dinkelbach's algorithm is used to solve the resulting mixed integer linear fractional programming model. The proposed framework is successfully applied to an industrial case study of a process to purify recombinant Fc Fusion protein from low molecular weight and high molecular weight product related impurities, involving two chromatographic steps with eight and three candidate resins for each step, respectively. The computational results show the advantage of the proposed framework in terms of computational efficiency and flexibility. © 2017 The Authors Biotechnology Progress published by Wiley Periodicals, Inc. on behalf of American Institute of Chemical Engineers *Biotechnol. Prog.*, 33:1116–1126, 2017

## Introduction

In the early stages of purification process development, different types of resins need to be tested at small scale (1.5–5000 µL) under various operating conditions, including different pH values, salt concentrations, and flow rates, to establish the resin most suited for process application at large scale.^1^ Platforms that have a capacity for high‐throughput screening (HTS) are commonly used to identify the most promising candidates for further investigation, in terms of key criteria of large scale purification, like yield, purity, and productivity.^2^, ^3^, ^4^, ^5^, ^6^, ^7^, ^8^, ^9^, ^10^, ^11^, ^12^ In HTS, the combination of robotic methods, parallel processing, and the miniaturization of bioprocess unit operations allows for a large number of potential process parameters to be examined within a short time, and also results in the generation of large amounts of data for evaluation. To deal with the substantial volume of data generated from such microscale HTS experiments, rapid analysis using a systematic methodology to focus on the conditions that result in optimal overall process performance can become therefore critical.

An additional concern is that current HTS methods optimize a chromatographic step irrespective of the rest of the chromatographic steps. Each microscale experiment is capable of being implemented for only a single resin, and hence the optimal resin is only the best one for the specific conditions tested in that experiment. However, in practice at industrial scale, a chromatography sequence, with two to four chromatographic separation steps, is usually implemented. Thus, the best resin for one separation step may not be the best choice when the whole sequence is considered, since performance is also related to the resins used at the other steps in the chromatographic sequence, their operating conditions and performance. It is critical to use a systematic approach to select promising resins for integrated chromatographic separations. In this work, we address the rapid selection of optimal resins for integrated chromatographic separations by proposing the use of mathematical programming techniques. The data generated by the HTS experiments are used as the inputs of the proposed approach to select the most promising resins for more tests in the following drug development stages. Note that the proposed approach does not affect how the HTS experiments are operated to generate the data, and it is assumed that these experiments are conducted following the standard protocol, and the data generated for the approach are accurately measured and examined.

Lately, the application of optimization‐based models, approaches, and tools in the biopharmaceutical industry have become more popular in the industry, e.g., production planning and scheduling,^13^, ^14^, ^15^ capacity planning,^16^ purification process synthesis,^17^, ^18^, ^19^, ^20^, ^21^, ^22^, ^23^, ^24^, ^25^ and downstream chromatography column sizing design using mathematical programming^26^, ^27^, ^28^, ^29^, ^30^, ^31^ and evolutionary algorithms.^32^, ^33^, ^34^, ^35^, ^36^ However, there is limited literature on resin selection for downstream purification processes. A three‐step approach was developed to screen resins for chromatographic optimization with an anion‐exchange chromatography column in the purification process.^37^ A model‐based rational strategy was proposed for the selection of chromatographic resins, in which multiple performance metrics, including yield, purity, productivity, resin/solvent cost, and concentration factor, were optimized using a genetic algorithm.^32^ This work was later extended to the selection of the most optimal process scheme from several possible alternatives.^33^ A series of mixed integer programming‐based models and approaches were developed for the downstream chromatography resin selection and sequencing strategies, integrated with chromatography column sizing decisions, of the downstream purification process of a monoclonal antibody (mAb).^28^, ^29^, ^30^ An evolutionary multiobjective optimization algorithm was developed for the optimal sequences of chromatographic purification steps and column sizing strategies considering multiple criteria.^35^

Recently, multiobjective mixed integer programming techniques were also applied to optimal resin selection for chromatographic sequence used for protein purification.^38^ However, this work ignored the mass balance between two consecutive chromatographic steps, due to the limited available data. In that case, the purity of a multi‐step process was considered as the average of them, which was not fully accurate. The work presented in this article aims to extend our previous studies by developing an optimization‐based, systematic decision support framework for the rapid selection of resins for integrated chromatographic separations. The mass balance constraints introduced to link different chromatography steps, and yield and purity are correctly calculated as the two objective functions. In addition, to overcome the nonlinearities in the proposed optimization model, a novel solution method was developed based on the factional programming techniques.

The reminder of this article is organized as follows: the optimization problem is first described. Then, a multiobjective mixed integer programming model is proposed, followed by the introduction of the solution approaches for the model. The case study is described, while its results are discussed. Finally, conclusions are drawn.

## Problem Statement

**Figure 1:**
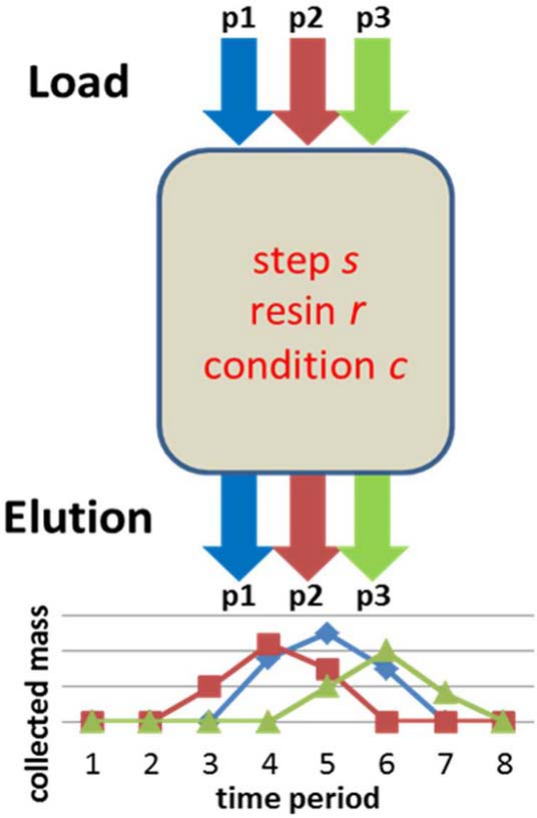
**Illustration of the HTS experiment.**

In a chromatographic purification process, target protein must be purified away from other impurity proteins using a resin selected from a set of potential candidates. A number of HTS microscale experiments are typically conducted, to select the most promising resins and their operating conditions from the candidates. Such an HTS experiment for a three‐protein mixture is illustrated in Figure 1. First, a mixture of proteins is loaded to a resin candidate under a specified range of conditions. The proteins are then collected at different time intervals by elution. Each resin is tested under different operating conditions, where each operating condition may refer to a unique and specific condition in pH and salt concentration. Different operating conditions could therefore have differences in either pH, or salt concentration, or both.

In the experiment for each resin and under each operating condition, gradient elution is typically implemented by changing eluent salt concentration across a fixed range.^39^ The whole elution process is divided into multiple time intervals. The mass of each protein in the eluate is determined. Beside the elution phase, the time intervals for each of the other phases in the separation are considered, including the load, wash, and regeneration steps. As the experiments are implemented only for single chromatography step, there is no data available for the synthesis of multiple chromatography steps. To overcome the problem of protein mass prediction from the limited data available in the purification process, and establish the links between consecutive two steps, it is assumed that for a specific protein, its mass collected at each time interval remains a constant ratio of its loaded mass, and is not affected by the loaded mass of other proteins. Thus, the data obtained from each HTS experiment can be used to calculate the actual mass collected, and the relationship of the mass collected at two consecutive steps are established. In this work, the starting and finishing time intervals for the target protein collection, which are linked to the salt concentration used in a gradient elution, are the variables to be optimized.

Overall, based on the data from the HTS experiments, we consider the resin selection problem as an optimization problem, which is described as follows:

*Given*:

- target protein, and impurity proteins;
- a purification process including multiple chromatographic steps;
- a number of chromatography resins for each chromatographic step;
- chromatography operating conditions of each resin;
- protein mass loaded under each condition for each resin;
- protein mass collected under each condition in each time interval for each resin;
- gradient eluent salt concentration in each time interval for each resin;

*to determine*:

- best resin at each step;
- best chromatography operating conditions;
- starting and finishing time for protein collection;

*so as to*: maximize key performance metrics of the chromatography sequence, including the yield and purity of the target protein.

## Mathematical Formulation

To solve the above optimization problem, we formulated a multiobjective mixed integer nonlinear programming (MINLP) model, which is presented in this section.

### Resin/operating condition

For each chromatographic separation step *s*, only one operating condition *c* of one resin *r* can be selected:

(1) $$\sum_{r\in R_{s}}\sum_{c\in C_{r}}Z_{src}=1,\;\forall s$$

where $Z_{src}$ is a binary variable which is equal to 1 if operating condition *c* of resin *r* is selected at step *s*.

### Collection time

At each chromatographic separation step *s*, there is only one operating starting cut‐point, as well as one finishing cut‐point, for protein collection:

(2) $$\sum_{t}{Xs}_{st}=1,\;\forall s$$

(3) $$\sum_{t}{Xf}_{st}=1,\;\forall s$$

where the binary variable ${Xs}_{st}$ indicates whether the beginning of time interval *t* is the starting time cut‐point at chromatographic separation step *s*; and ${Xf}_{st}$ indicates whether the end of time interval *t* is the finishing time cut‐point at chromatographic separation step *s*.

**Figure 2:**
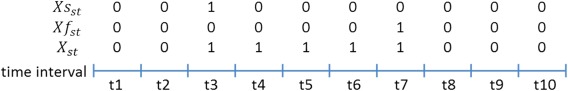
**Example of time interval selection.**

When the values of variables ${Xs}_{st}$ and ${Xf}_{st}$ are known, the values of binary variables $X_{st}$ for the decision whether time interval *t* is selected for protein collection at chromatographic separation step *s* can be derived. As illustrated in Figure 2, for an example of ${Xs}_{s,t3}={Xf}_{s,t7}=1$, the value of $X_{st}$ equals 1 for time intervals t4, t5, t6, and t7.

Thus, the time intervals selected for protein collection are determined as follows:

(4) $$X_{st}={\left.X_{s,t-1}\right|}_{t>1}+{Xs}_{st}-{\left.{Xf}_{s,t-1}\right|}_{t>1},\;\forall s,t$$

Next, we define a binary variable $W_{srct}$ to indicate whether time interval *t* is selected for collection under condition *c* of resin *r* at step *s*, which is equivalent to the product of three binary variables defined above, $X_{st}$ and $Z_{src}$. Thus, $W_{srct}=1$, if time interval *t* is selected for resin *r* under condition *c* at step *s*. If either one of $X_{st}$ and $Z_{src}$ is zero, $W_{srct}$ is zero. Thus, we have the following Eqs. (5) and (6):

(5) $$\sum_{r\in R_{s}}\sum_{c\in C_{r}}W_{srct}\leq U\cdot X_{st},\;\forall s,t$$

(6) $$\sum_{t}W_{srct}\leq U\cdot Z_{src},\;\forall s,r\in R_{s},c\in C_{r}$$

where parameter *U* is a large number. In addition, if $X_{st}$ and $Z_{src}$ are both equal to one, then $W_{srct}$ becomes one. Therefore, we have the following constraint:

(7) $$W_{srct}\geq X_{st}+Z_{src}-1,\;\forall s,r\in R_{s},c\in C_{r},t$$

### Mass balance

In the first step ( fs) of the chromatographic separation process, the loaded protein mass is the same as that loaded in the experiment using the selected resin and operating condition.

(8) $${LM}_{sp}=\sum_{r\in R_{s}}\sum_{c\in C_{r}}{lm}_{scrp}\cdot Z_{scr},\;\forall s=fs,p$$

where ${lm}_{srcp}$ is the loaded mass of protein *p* under operating condition *c* of resin *r* at chromatographic separation step *s* in the HTS experiment.

Similarly, the collected protein mass at the first step is also the same as the experiment data.

(9) $$M_{srcpt}={cm}_{srcpt}\cdot W_{srct},\;\forall s=fs,r\in R_{s},c\in C_{r},p,t$$

where ${cm}_{srcpt}$ is the collected mass of protein *p* under operating condition *c* of resin *r* in time interval *t* at chromatographic separation step *s* in the HTS experiment.

The total mass of protein *p* collected in all time periods at step *s*, ${CM}_{sp}$, is also the mass for loading at step *s* + 1, ${LM}_{s+1,p}$, as defined below:

(10) $${CM}_{sp}=\sum_{r\in R_{s}}\sum_{c\in C_{r}}\sum_{t}M_{srcpt},\;\forall s,p$$

(11) $${LM}_{s+1,p}={CM}_{sp},\;\forall s,p$$

**Figure 3:**
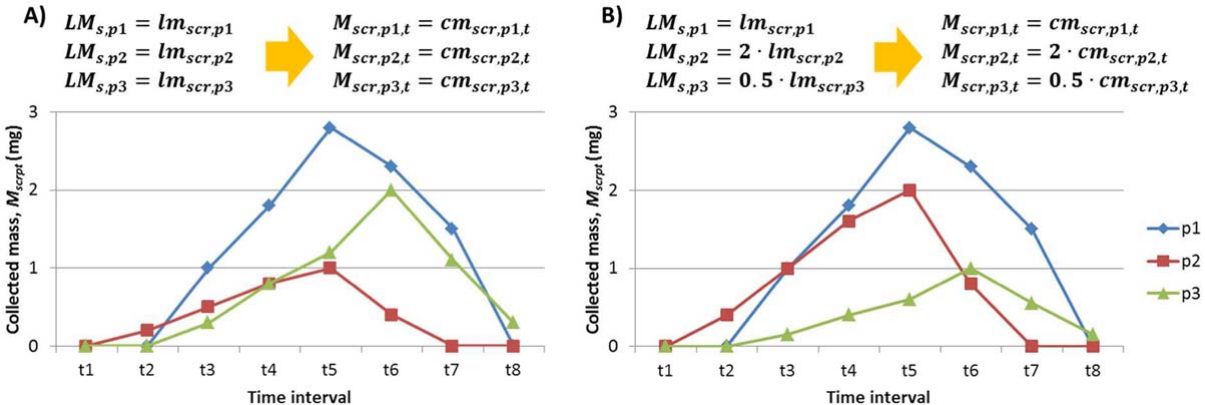
**Examples of the calculation of the collected protein mass.**

However, for the chromatography steps rather than the first one in the chromatography purification process, the protein mass amount loaded to the others steps of the chromatography process, which is the amount of mass collected in the previous step, may not have be implemented in the experiments. Facing the limited available data, to predict the collected protein mass in the proposed optimization model, it is assumed that the mass of protein collected in each time period is proportional to the loaded mass, and the ratio is constant and not affected by the loaded mass of other proteins. Let ${lcr}_{srcpt}=\frac{{cm}_{srcpt}}{{lm}_{srcp}}$ be the constant ratio of the collected mass to the loaded mass of each protein in the HTS experiments, which is derived from the single‐step experiment data. Thus, for the selected resin *s* and operating condition *c*, the collected protein mass is calculated by the loaded mass multiplied by the constant ratio, i.e., $M_{srcpt}={lcr}_{srcpt}\cdot {LM}_{sp}=\frac{{LM}_{sp}}{{lm}_{srcp}}\cdot {cm}_{srcpt}$ if $W_{srcpt}=1$. Figure 3 presents two examples of the collected protein mass calculation of a mixture of three proteins (p1–p3) based on the above assumption, in which examples A and B have different loaded protein mass. In example A, the loaded mass is the same as that in the single‐step experiment, i.e., ${LM}_{sp}={lm}_{srcp}$. Then according to the assumption, the collected protein mass is equal to that in the single‐step experiment, i.e., $M_{srcpt}={cm}_{srcpt}$. In example B, the loaded protein mixture has more protein p2 and less protein p3. Specifically, compared to example A, the loaded mass of protein p2 is doubled, while that of protein p3 is halved. Thus, according to the assumption of constant collection/load ratio, the collected mass of protein p2 also becomes twice that in example A ( $M_{srcpt}=2\cdot {cm}_{srcpt}$), and that of p3 becomes only half of example A $\left(M_{srcpt}=0.5\cdot {cm}_{srcpt}\right)$.

Thus, the following proposed constraints are proposed to enforce the assumption that the collected protein mass, $M_{srcpt}$, is equal to the loaded mass, ${LM}_{sp}$, multiplied by the known ratio, ${lcr}_{srcpt}$, only when the corresponding resin/condition/time combination is selected, i.e., $W_{srct}=1$; otherwise, no protein is collected under that condition:

(12) $$M_{srcpt}\leq {lcr}_{srcpt}\cdot {LM}_{sp},\;\forall s\neq fs,r\in R_{s},c\in C_{r},p,t$$

(13) $$M_{srcpt}\geq {lcr}_{srcpt}\cdot {LM}_{sp}-U\cdot \left(1-W_{srct}\right),\;\forall s\neq fs,r\in R_{s},c\in C_{r},p,t$$

(14) $$M_{srcpt}\leq U\cdot W_{srct},\;\forall s\neq fs,r\in R_{s},c\in C_{r},p,t$$

### Salt concentration linking

The salt concentration of eluent at the former step is required to be no more than that at the later step in the multi‐step process. Thus, the salt concentration at the selected finishing cut‐point at step *s* − 1 is less than or equal to that of the starting cut‐point at step *s*:

(15) $$\sum_{t}{sc}_{s-1,t}\cdot {Xf}_{s-1,t}\leq \sum_{t}{sc}_{s,t}\cdot {Xs}_{s,t},\;\forall s\neq fs$$

where ${sc}_{st}$ is the corresponding salt concentration of time interval *t* at chromatographic separation step *s*.

### Objective functions

For the chromatographic separation process, we consider two performance criteria, which are yield and purity. The yield, Y, is defined as the ratio of the collected mass of the target protein, *dp*, at the last step to its loaded mass at the first step:

(16) $$Y=\frac{{CM}_{ls,dp}}{{LM}_{fs,dp}}$$

The overall purity of target protein, P, is the ratio of the collected mass of the target protein, *dp*, to that of all proteins:

(17) $$P=\frac{{CM}_{ls,dp}}{\sum_{p}{CM}_{ls,p}}$$

### Multiobjective optimization problem

With the above constraints, the multiobjective optimization problem was formulated as an MINLP model in the following format.

(18) max⁡ {Y,P}

s.t. Eqs. (1)–(17)

## Solution Approaches

The classic *ε*‐constraint method was applied to the multiobjective optimization problem. The developed MINLP model was transformed into a mixed integer linear fractional programming (MILFP) model for locating the Pareto optimum. Then, to solve the resulting MILFP model, the Dinkelbach's algorithm was used to solve a set of mixed integer linear programming (MILP) models. In this section, we first introduce the classic *ε*‐constraint method. Then, the Dinkelbach's algorithm is briefly described.

### ε‐constraint method for multiobjective optimization

To solve the above multiobjective optimization problem (18), we applied the classic *ε*‐constraint method,^40^, ^41^ which has been widely used in the literature for mutiobjective optimization.^42^, ^43^, ^44^ In this method, all but one objective are converted into constraints by setting an upper or lower bound to each of them, and only one objective is optimized. Thus, for each specific value of ε, the multiobjective optimization problem (18) can be transformed into a single objective optimization problem (19) by maximizing the yield only and converting the purity into constraints:

$$\max\;\frac{{CM}_{ls,dp}}{{LM}_{fs,dp}}$$

(19) $$s.t.\;{CM}_{ls,dp}\geq \varepsilon \cdot \sum_{p}{CM}_{ls,p}$$

Eqs. (1)–(15)

where ε in this case is the minimum requirement of the purity. The above single‐objective optimization model (19) involves linear constraints and a fractional objective function with both numerator and denominator as linear functions. Thus, the above developed optimization model (19) is an MILFP model. For a special case of the same protein mass loaded in each experiment, the objective function in the optimization model (19) developed becomes a linear function by replacing variable ${LM}_{fs,dp}$ by a parameter for the constant loaded protein mass, and therefore the model (19) is an MILP model.

**Figure 4:**
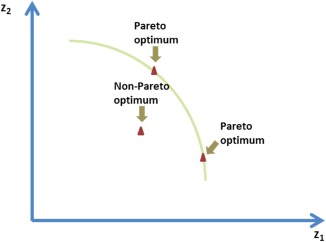
**Pareto‐optimal solutions in bi‐objective optimization.**

In most cases, the objective functions in the multiobjective optimization problems conflict with each other, and there exists no solution which can optimize all objective functions simultaneously. Thus, the solutions of a multiobjective problem are generated as Pareto‐optimal solutions.^45^ The Pareto‐optimal solution of a multiobjective problem is the one such that no other solution can be better in one objective without being worse in any one of other objectives. The Pareto‐optimal solutions of a bi‐objective optimization problem are shown in Figure 4. The weak Pareto‐optimal solution of a multiobjective problem is the one such that no other solution can be better in all objectives.

The Pareto optimality of the solutions of the problem (18) follows from the Theorems 1 and 2, whose proof can be found in the literature^46^:

Theorem 1 $x^{*}$ *is Pareto‐optimal solution of multiobjective optimization problem (18), if it is a unique optimal solution of the optimization problem (19) for any given lower bound,* ε.

Theorem 2 $x^{*}$ *is weak Pareto‐optimal solution of multiobjective optimization problem (18), if it is an optimal solution of the optimization problem (19) for any given lower bound,* ε.

### Dinkelbach's algorithm for MILFP model

To solve the MILFP model (19) in the *ε*‐constraint method, we implemented the Dinkelbach's algorithm. The Dinkelbach's algorithm is an application of the classical Newton method to solve convex nonlinear fractional programming models by solving a sequence of nonlinear programming (NLP) models successively.^47^ Recently, it has been widely used to solve MILFP problems.^29^, ^30^, ^48^, ^49^, ^50^, ^51^, ^52^, ^53^

The Dinkelbach's algorithm iteratively solves an MILP model, by reformulating the objective function of the MILFP model as a linear function, instead of solving the MILFP model directly. This is achieved by solving the model with an updated parameter in the linear objective function in each iteration, until the termination criterion is met, i.e., the objective value of the MILP model is close enough to zero within a given tolerance of δ. The MILFP model (19) is first transformed into the corresponding MILP model, by reformulating the original objective function, as follows:

$$\max\;{CM}_{ls,dp}-f\cdot {LM}_{fs,dp}$$

(20) $$s.t.\;{CM}_{ls,dp}\geq \varepsilon \cdot \sum_{p}{CM}_{ls,p}$$

Eqs. (1)–(15)

where f is a parameter whose value keeps updated during the iterations. The Dinkelbach's algorithm procedure is described as below:

**Step 1**: Initialize f;

**Step 2**: Solve the MILP model (20), and the obtained values of ${CM}_{sp}$ and ${LM}_{sp}$ in the solutions are denoted as ${CM}_{sp}^{*}$ and ${LM}_{sp}^{*}$, respectively;

**Step 3**: If $\;\left|{CM}_{ls,dp}^{*}-f\cdot {LM}_{fs,dp}^{*}\right|\leq \delta$, stop; otherwise, update $=\frac{{CM}_{ls,dp}^{*}}{{LM}_{fs,dp}^{*}}$, then go to Step 2.

**Figure 5:**
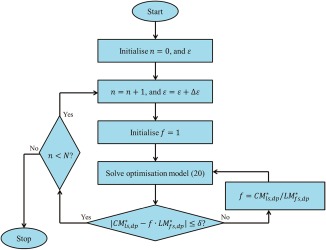
**Solution procedure.**

Thus, to solve the developed multiobjective MINLP model, a series of MILP models were solved iteratively. The whole solution procedure is illustrated in Figure 5, combining both the *ε*‐constraint method and Dinkelbach's algorithm, in which *N* values of *ε* with a step of *Δε* were implemented in the *ε*‐constraint method.

## Case Study

Here, we consider a real case study of a biopharmaceutical company, wishing to purify recombinant Fc Fusion protein (monomer) from low molecular weight and high molecular weight product related impurities (aggregates and fragments). The loaded mixture of proteins keep the same proportion of components, including 86.2% of monomer, 10.6% of aggregates, and 3.2% of fragments. A two‐step chromatography sequence comprises cation‐exchange chromatography (CEX) as the first step and mixed mode chromatography (MM) as the second one. The microscale experiments were implemented only for one single step, using 600 µL Robo Columns (Repligen, USA), which were operated using a Tecan Evo 200 liquid handling robot. Purity was determined using a TSK‐Gel G3000 column (Tosoh Biosciences, Japan) coupled to an Agilent HPLC Series 1200 HPLC (Agilent, UK). Protein was quantified using a Trinean Dropsense 16 micro‐volume spectroscope (Trinean, Belgium). It needs to be noted that the amount of other impurities, e.g., host cell protein (HCP) and deoxyribonucleic acid (DNA), was low already and therefore not included in the analysis.

The CEX and MM candidate resins investigated were all commercially available and were selected for evaluation based on their different chemical characteristics and purification capabilities. For CEX, there were eight candidate resins with two operating conditions (each referring to a running pH value used) for each resin, while for MM, there were three candidate resins with from 7 to 12 operating conditions (each referring to a specific combination of a running pH value and a loading salt concentration level) for each resin. For reasons of confidentiality, the detailed operating conditions are not revealed in this article. The labels in Table 1 are used to represent the candidate resins and their operating conditions.

**Table 1:** Candidate Resins and Operating Conditions

Step	Resin name	Resin label	Operating condition label
CEX	Catpo Impres	RCEX1	CCEX1‐1, CCEX1‐2
	Capto S	RCEX2	CCEX2‐1, CCEX2‐2
	Poros XS	RCEX3	CCEX3‐1, CCEX3‐2
	Poros HS 50	RCEX4	CCEX4‐1, CCEX4‐2
	Nuvia S	RCEX5	CCEX5‐1, CCEX5‐2
	Toyopearl	RCEX6	CCEX6‐1, CCEX6‐2
	S Hypercel	RCEX7	CCEX7‐1, CCEX7‐2
	Fractogel	RCEX8	CCEX8‐1, CCEX8‐2
MM	PPA Hypercel	RMM1	CMM1‐1, CMM1‐2, CMM1‐3, CMM1‐4, CMM1‐5, CMM1‐6, CMM1‐7
	HEA Hypercel	RMM2	CMM2‐1, CMM2‐2, CMM2‐3, CMM2‐4, CMM2‐5, CMM2‐6, CMM2‐7, CMM2‐8
	Nuvia	RMM3	CMM3‐1, CMM3‐2, CMM3‐3, CMM3‐4, CMM3‐5, CMM3‐6, CMM3‐7, CMM3‐8, CMM3‐9, CMM3‐10, CMM3‐11, CMM3‐12

The whole purification process at each step was divided into a number of time intervals. The corresponding phase and salt concentration of each interval are given in Table 2. Note that the salt concentration gradients are different in the CEX and MM chromatographic modes. The CEX chromatographic separation experiment employed salt concentration gradients from 0 to 300 mM over 17 time intervals (T1–T17). It operated in bind‐elute mode. The flow‐through MM chromatographic separation experiment use salt concentration gradients from 0 to 1000 mM, and 29 time intervals (T1–T29) for the load, wash, elution, and regeneration steps. The collected protein mass of monomer, aggregate, and fragment, in each time interval of each experiment was determined experimentally.

**Table 2:** Phases, Time Intervals, and Sodium Chloride Gradients in Chromatographic Separation

Phase		Load	Wash		Elution						
Interval		T1	T2	T3	T4	T5	T6	T7	T8	T9	T10
[Sodium chloride]	CEX	–	–	–	0	0	50	50	100	100	150
(mM)	MM	–	–	–	0	0	50	50	100	100	200
Phase		Elution									
Interval		T11	T12	T13	T14	T15	T16	T17	T18	T19	T20
[Sodium chloride]	CEX	150	200	200	250	250	300	300	–	–	–
(mM)	MM	200	300	300	400	400	500	500	600	600	700
Phase		Elution							Regeneration		
Interval		T21	T22	T23	T24	T25	T26	T27	T28	T29	
[Sodium chloride]	CEX	–	–	–	–	–	–	–	–	–	
(mM)	MM	700	800	800	900	900	1000	1000	–	–	

The developed optimization framework was implemented in GAMS 24.4^54^ on a Microsoft Windows 7 based machine with Intel Xeon W3670 3.2 GHz processor and 12 GB RAM, using MILP CPLEX solver. The optimality gap, i.e., termination tolerance for use in solving MILP models, was set to 0%, to guarantee that the solution process only stops when the solution found is the best theoretical objective value.

## Results

The developed model was applied to each of the two separation steps independently at first, and then to the integrated two‐step separation process. In the *ε*‐constraint method implemented in this work, we used 10 values of *ε* increasing from 90 to 99% by a step of 1%. The results obtained are discussed and analyzed later in this section.

### CEX chromatographic separation

**Figure 6:**
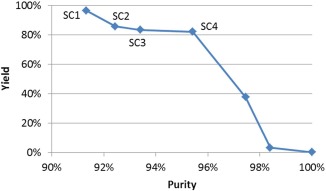
**Pareto frontier of CEX chromatographic separation.**

First, we selected the resins and conditions for the CEX step in the Pareto‐optimal solutions. Using the Dinkelbach's algorithm, each MILFP was solved by solving 2–4 MILP models, each of which took less than 1 s to locate the optimal solution. Figure 6 shows the obtained Pareto frontiers of the CEX chromatographic separation. There are four Pareto‐optimal solutions (SC1–SC4) both having high yield (over 80%) and purity (over 90%). A higher purity of over 96% is however only achievable at a very high cost of sacrificing yield. The details of the four Pareto optimal solutions are given in Table 3. Here, different resins were selected to meet the different yield requirements. Resins RCEX1, RCEX3, and RCEX8 were the most promising and were selected for further investigation.

**Table 3:** Pareto‐Optimal Solutions of CEX Chromatographic Separation

Solution	Resin	Condition	Collection phase and time interval	Yield (%)	Purity (%)
SC1	RCEX3	CCEX3‐1	Elution (0–250 mM NaCl): T4–T15	96.6	91.3
SC2	RCEX1	CCEX1‐1	Elution (0–150 mM NaCl): T4–T11	85.9	92.4
SC3	RCEX8	CCEX8‐1	Elution (0–200 mM NaCl): T4–T13	83.6	93.4
SC4	RCEX8	CCEX8‐1	Elution (200 mM NaCl): T12–T13	82.2	95.4

### MM chromatographic separation

**Figure 7:**
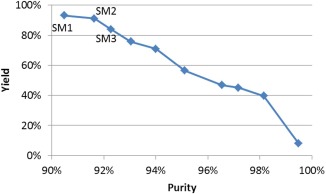
**Pareto frontier of MM chromatographic separation.**

Considering only the MM separation step, 10 Pareto‐optimal solutions were found in 12 s of computation, as shown in Figure 7. Here, three of them (SM1–SM3) with both high yield (over 80%) and purity (over 90%) are highlighted and presented in Table 4. In this case, the resin RMM3 with its operating condition CMM3‐10 had a dominating performance. Although the same resin/condition combination was selected for different minimum purity requirements, the time intervals for protein collection had to vary to achieve higher purities.

**Table 4:** Pareto‐Optimal Solutions of MM Chromatographic Separation

Solution	Resin	Condition	Collection phase and time interval	Yield (%)	Purity (%)
SM1	RMM3	CMM3‐10	Load, wash, elution (0–300 mM NaCl): T1–T12	93.1	90.5
SM2	RMM3	CMM3‐10	Load, wash, elution (0–200 mM NaCl): T1–T11	91.1	91.6
SM3	RMM3	CMM3‐10	Wash, elution (0–200 mM NaCl): T3–T11	84.0	92.3

### Integrated chromatographic separations

**Figure 8:**
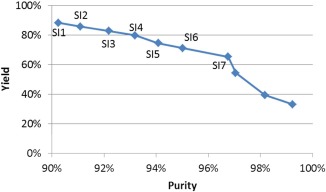
**Pareto frontier of integrated CEX‐MM chromatographic separation.**

Next, we considered the integration of the above two steps, including CEX as the first step and MM as the second one. Figure 8 shows the nine Pareto‐optimal solutions found by the ε‐constraint method. It can be observed that while there is no solution with a yield of over 90% for the two‐step separations, high purities are still achievable. For each value of ε, 2–4 iterations of the Dinkelbach's algorithm taking a total computational time of up to 3 min were required.

Table 5 shows the details of the seven Pareto‐optimal solutions with higher yields (>60%). All chromatographic separation sequences were dominated by the sequences of RCEX1‐RMM3 and RCEX3‐RMM3. Comparing with single‐step CEX separation, the resin RCEX1 was the most promising for obtaining higher yields at the CEX step, while RCEX3 was selected to achieve high purity. Both of the aforementioned two are also selected for the single‐step CEX separation. However, different operation conditions, CCEX1‐2 and CCEX3‐2, were selected for resins RCEX1 and RCEX3, respectively, rather than conditions CCEX1‐1 and CCEX3‐1 used for the single‐step CEX separation. This means that the elution pH was increased to reduce the amount of salt required to elute the product at the CEX step and enable compatibility with the subsequent MM step. Similarly, for resin RMM3, different conditions CMM3‐1 and CMM3‐5 were selected for the integrated separation, CMM3‐10 selected for the single‐step MM separation. The optimal protein collection time intervals for the CEX step were from T6 to T7 for resin RCEX1, and from T4 to T9 for resin RCEX3 at different Pareto‐optimal solutions, while those at the MM step were adjusted to achieve higher purities greater than 90%. In addition, it can be seen that for the integrated process with two steps, for similar obtained purity after purification, the yield of the process is much lower than the single step. Thus, when the higher yield is preferred, single step process will be a better choice. Note that it is valid without considering the process capability to clear other impurities, e.g., virus, HCP, DNA.

**Table 5:** Pareto‐Optimal Solutions of CEX‐MM Chromatographic Separation

Solution	Resin	Condition	Collection phase and time interval	Yield (%)	Purity (%)
SI1	RCEX1 RMM3	CCEX1‐2 CMM3‐1	CEX–Elution (50 mM NaCl): T6–T7 MM–Elution (50–1000 mM NaCl): T6–T27	88.4	90.2
SI2	RCEX1 RMM3	CCEX1‐2 CMM3‐1	CEX–Elution (50 mM NaCl): T6–T7 MM–Elution (50–700 mM NaCl): T6–T20	85.8	91.1
SI3	RCEX1 RMM3	CCEX1‐2 CMM3‐1	CEX–Elution (50 mM NaCl): T6–T7 MM–Elution (50–300 mM NaCl): T6–T12	82.9	92.2
SI4	RCEX1 RMM3	CCEX1‐2 CMM3‐1	CEX–Elution (50 mM NaCl): T6–T7 MM–Elution (50–200 mM NaCl): T6–T11	79.8	93.2
SI5	RCEX1 RMM3	CCEX1‐2 CMM3‐10	CEX–Elution (50 mM NaCl): T6–T7 MM–Elution (50–300 mM NaCl): T6–T12	74.7	94.1
SI6	RCEX3 RMM3	CCEX3‐2 CMM3‐5	CEX–Elution (0–100 mM NaCl): T4–T9 MM–Elution (100–400 mM NaCl): T8–T14	71.2	95.0
SI7	RCEX3 RMM3	CCEX3‐2 CMM3‐5	CEX–Elution (0–100 mM NaCl): T4–T9 MM–Elution (100–200 mM NaCl): T8–T11	65.3	96.8

**Figure 9:**
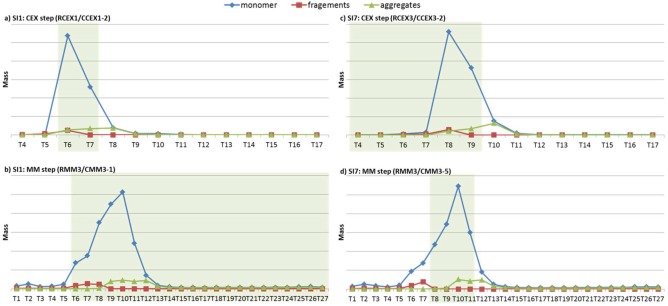
**Chromatograms and the optimal cut‐points for protein collection in Pareto‐optimal solutions SI1 and SI7.**

Here, the two Pareto‐optimal solutions, SI1 and SI7, are focused for further discussion in Figure 9. In solution SI1, resin RCEX1 and condition CCEX1‐2 are selected at the CEX step, and resin RMM3 and condition CMM3‐1 are the optimal choice at the MM step. To achieve high yield, most of the time periods with high monomer mass are selected. While, in solution SI7, a different resin (RCEX3) is selected for the CEX step in solution SI7, and another condition (CMM3‐5) is used of resin RMM3 at the MM step. Several time intervals with high monomer mass are not selected within the starting and finishing cut‐points to avoid peaks of the impurities. It can be seen that the achieved yield is much sacrificed to achieve high purity, which demonstrates the trade‐off between yield and purity.

## Conclusion

In this work, we developed an optimization‐based decision support systematic framework for the problem of rapid resin selection when seeking an optimized, integrated chromatographic separation. A multiobjective mixed integer programming model has been developed to maximize both yield and purity of both single‐step and integrated multi‐step chromatographic separations. The classic *ε*‐constraint method was adopted as the solution approach, in which only yield was optimized subject to the purity requirement constraints. The resulting single‐objective MILFP model in the *ε*‐constraint method was solved by the established Dinkelbach's algorithm. The developed framework was applied to a real case study to show its applicability. The results show that the proposed method can process a huge amount of experimental data, and identify the best resins within a few minutes of computational time.

Compared to manual comparison and decision making which is the current practice of the industry, one significant benefit of the proposed systematic framework lie additionally in the computational efficiency. In addition, the developed decision framework is quite generic and flexible, and has the advantage of being able to accommodate different case studies and datasets. There still exist some limitations of this work. The model developed in this work is based on the assumption that the ratio of the collected protein mass to the loaded protein remains constant. However, the further investigation will need to be taken in the future research to see how this assumption is affected by the experiment conditions, such as the loading protein concentrations, pH values, flow linear velocities, etc. In addition, another key future work direction is the verification of the scale‐up processes to validate the obtained solutions in larger scale experiments.

## Notation

### Indices

*c*: operating condition
*dp*: target protein
p: protein, including target protein and impurities
*fs*: first chromatographic step in the process
*ls*: last chromatographic step in the process
*r*: resin
*s*: chromatographic step
*t*: time interval

### Sets

$C_{r}$: set of conditions available to resin *r*
$R_{s}$: set of resins available at chromatographic step *s*

### Parameters

${cm}_{srcpt}$: mass of protein *p* collected under condition *c* of resin *r* in time interval *t* at chromatographic step *s* in single‐step HTS experiment, mg
${lcr}_{srcpt}$: ratio of mass of protein *p* collected in time interval *t* to that loaded under condition *c* of resin *r* at chromatographic step *s* in single‐step HTS experiment
${lm}_{srcp}$: mass of protein *p* loaded under condition *c* of resin *r* at chromatographic step *s*, in single‐step HTS experiment, mg
${sc}_{st}$: salt concentration during the elution in time interval *t* at chromatographic step *s*, mM
U: a large number

### Continuous variables

${CM}_{sp}$: total mass of protein *p* collected at chromatographic step *s*, mg
${LM}_{sp}$: mass of protein *p* loaded at chromatographic step *s*, mg
$M_{srcpt}$: mass of protein *p* collected under condition *c* of resin *r* in time interval *t* at chromatographic step *s*, mg
P: overall purity of target protein
Y: overall yield of target protein

### Binary variables

$W_{srct}$: 1 if time interval *t* of resin *r* under condition *c* is selected at chromatographic step *s*; 0 otherwise
$X_{st}$: 1 if time interval *t* is selected at chromatographic step *s*; 0 otherwise
${Xf}_{st}$: 1 if end of time interval *t* is the finishing time cut‐point at chromatographic step *s*; 0 otherwise
${Xs}_{st}$: 1 if beginning of time interval *t* is the starting time cut‐point at chromatographic step *s*; 0 otherwise
$Z_{src}$: 1 if condition c of resin *r* is selected at chromatographic step *s*; 0 otherwise

## References

[1] Jagschies G , Sofer GK , Hagel L. Handbook of Process Chromatography: Development, Manufacturing, Validation and Economics, 2nd ed London, UK: Academic Press; 2008.

[2] Mazza CB , Rege K , Breneman CM , Sukumar N , Dordick JS , Cramer SM. High‐throughput screening and quantitative structure‐efficacy relationship models of potential displacer molecules for ion‐exchange systems. Biotechnol Bioeng. 2002;80:60–72. 12209787 10.1002/bit.10343

[3] Bensch M , Schulze Wierling P , von Lieres E , Hubbuch J. High throughput screening of chromatographic phases for rapid process development. Chem Eng Technol. 2005;28:1274–1284.

[4] Charlton H , Galarza B , Beacon B , Leriche K , Jones R. Chromatography process development using 96‐well microplate formats. BioPharm Int. 2006;June suppl:20–26.

[5] Wenger MD , DePhillips P , Price CE , Bracewell DG. An automated microscale chromatographic purification of virus‐like particles as a strategy for process development. Biotechnol Appl Biochem. 2007;47:131–139. 17311568 10.1042/BA20060240

[6] Coffman J , Kramarczyk J , Kelley B. High‐throughput screening of chromatographic separations: I. Method development & column modeling. Biotechnol Bioeng. 2008;100:605–618. 18496874 10.1002/bit.21904

[7] Teeters M , Bezila D , Alred P , Velayudhan A. Development and application of an automated, low‐volume chromatography system for resin and condition screening. Biotechnol J. 2008;3:1212–1223. 18543245 10.1002/biot.200700265

[8] Boushaba R , Baldascini H , Gerontas S , Titchener‐Hooker NJ , Bracewell DG. Demonstration of the use of windows of operation to visualize the effects of fouling on the performance of a chromatographic step. Biotechnol Prog. 2011;27:1009–1017. 21567992 10.1002/btpr.617

[9] Connell‐Crowley L , Larimore EA , Gillespie R. Using high throughput screening to define virus clearance by chromatography resins. Biotechnol Bioeng. 2013;110:1984–1994. 23436296 10.1002/bit.24869

[10] Close EJ , Salm JR , Bracewell DG , Sorensen E. A model based approach for identifying robust operating conditions for industrial chromatography with process variability. Chem Eng Sci. 2014;116:284–295.

[11] Lacki KM. High throughput process development in biomanufacturing. Curr Opin Chem Eng. 2014;6:25–32.

[12] Traylor SJ , Xu X , Li Y , Jin M , Li ZJ. Adaptation of the pore diffusion model to describe multi‐addition batch uptake high‐throughput screening experiments. J Chromatogr A. 2014;1368:100–106. 25311484 10.1016/j.chroma.2014.09.058

[13] Lakhdar K , Savery J , Papageorgiou LG , Farid SS. Multiobjective long‐term planning of biopharmaceutical manufacturing facilities. Biotechnol Prog. 2007;23:1383–1393. 17924645 10.1021/bp0701362

[14] Lakhdar K , Zhou YH , Savery J , Titchener‐Hooker NJ , Papageorgiou LG. Medium term planning of biopharmaceutical manufacture using mathematical programming. Biotechnol Prog. 2005;21:1478–1489. 16209554 10.1021/bp0501571

[15] Liu S , Yahia A , Papageorgiou LG. Optimal production and maintenance planning of biopharmaceutical manufacturing under performance decay. Ind Eng Chem Res. 2014;53:17075–17091.

[16] Siganporia CC , Ghosh S , Daszkowski T , Papageorgiou LG , Farid SS. Capacity planning for batch and perfusion bioprocesses across multiple biopharmaceutical facilities. Biotechnol Prog. 2014;30:594–606. 24376262 10.1002/btpr.1860 PMC4415584

[17] Asenjo JA , Herrera L , Byrne B. Development of an expert system for selection and synthesis of protein purification processes. J Biotechnol. 1989;11:275–298.

[18] Vasquez‐Alvarez E , Lienqueo ME , Pinto JM. Optimal synthesis of protein purification processes. Biotechnol Prog. 2001;17:685–696. 11485430 10.1021/bp010031d

[19] Vasquez‐Alvarez E , Pinto JM. A mixed integer linear programing model for the optimal synthesis of protein purification processes with product loss. Chem Biochem Eng Q. 2003;17:77–84.

[20] Vasquez‐Alvarez E , Pinto JM. Efficient MILP formulations for the optimal synthesis of chromatographic protein purification processes. J Biotechnol. 2004;110:295–311. 15163520 10.1016/j.jbiotec.2004.02.009

[21] Simeonidis E , Pinto JM , Lienqueo ME , Tsoka S , Papageorgiou LG. MINLP models for the synthesis of optimal peptide tags and downstream protein processing. Biotechnol Prog. 2005;21:875–884. 15932268 10.1021/bp049650n

[22] Natali JM , Pinto JM , Papageorgiou LG. Efficient MILP formulations for the simultaneous optimal peptide tag design and downstream processing synthesis. AIChE J. 2009;55:2303–2317.

[23] Polykarpou EM , Dalby PA , Papageorgiou LG. Optimal synthesis of chromatographic trains for downstream protein processing. Biotechnol Prog. 2011;27:1653–1660. 21976368 10.1002/btpr.670

[24] Polykarpou EM , Dalby PA , Papageorgiou LG. A novel efficient optimisation system for purification process synthesis. Biochem Eng J. 2012;67:186–193.

[25] Polykarpou EM , Dalby PA , Papageorgiou LG. An MILP formulation for the synthesis of protein purification processes. Chem Eng Res Des. 2012;90:1262–1270.

[26] Liu S , Simaria AS , Farid SS , Papageorgiou LG. Mixed integer optimisation of antibody purification processes In: Kraslawski A, Turunen I., editors. 23rd European Symposium on Computer Aided Process Engineering, Computer Aided Chemical Engineering, Vol. 32 Amsterdam: Elsevier; 2013:157–162.

[27] Liu S , Simaria AS , Farid SS , Papageorgiou LG. Designing cost‐effective biopharmaceutical facilities using mixed‐integer optimization. Biotechnol Prog. 2013;29:1472–1483. 23956206 10.1002/btpr.1795

[28] Liu S , Simaria AS , Farid SS , Papageorgiou LG. An optimisation‐based approach for biopharmaceutical manufacturing In: Klemes JJ, Varbanov PS, Liew PY, editors. 24th European Symposium on Computer Aided Process Engineering, Computer Aided Chemical Engineering, Vol. 33 Amsterdam: Elsevier; 2014:1183–1188.

[29] Liu S , Simaria AS , Farid SS , Papageorgiou LG. Optimising chromatography strategies of antibody purification processes by mixed integer fractional programming techniques. Comput Chem Eng. 2014;68:151–164.

[30] Liu S , Simaria AS , Farid SS , Papageorgiou LG. Mathematical programming approaches for downstream processing optimisation of biopharmaceuticals. Chem Eng Res Des. 2015;94:18–31.

[31] Liu S , Farid SS , Papageorgiou LG. Integrated optimisation of upstream and downstream processing in biopharmaceutical manufacturing under uncertainty: A chance constrained programming approach. Ind Eng Chem Res. 2016;55:4599–4612.

[32] Nfor BK , Zuluaga DS , Verheijen PJT , Verhaert PDEM , van der Wielen LAM , Ottens M. Model‐based rational strategy for chromatographic resin selection. Biotechnol Prog. 2011;27:1629–1643. 22238769 10.1002/btpr.691

[33] Nfor BK , Ahamed T , van Dedem GWK , Verhaert PDEM , van der Wielen LAM , Epink MHM , van de Sandt EJAX , Ottens M. Model‐based rational methodology for protein purification process synthesis. Chem Eng Sci. 2013;89:185–195.

[34] Simaria AS , Turner R , Farid SS. A multi‐levelmeta‐heuristic algorithm for the optimisation of antibody purification processes. Biochem Eng J. 2012;69:144–154.

[35] Allmendinger R , Simaria AS , Farid SS. Multiobjective evolutionary optimization in antibody purification process design. Biochem Eng J. 2014;91:250–264.

[36] Allmendinger R , Simaria AS , Turner R , Farid SS. Closed‐loop optimization of chro‐matography column sizing strategies in biopharmaceutical manufacture. J Chem Technol Biotechnol. 2014;89:1481–1490. 25506115 10.1002/jctb.4267 PMC4258073

[37] Rathore AS. Resin screening to optimize chromatographic separations. LC GC N Am. 2001;19:616–622.

[38] Liu S , Gerontas S , Gruber D , Turner R , Titchener‐Hooker NJ , Papageorgiou LG. Optimal resin selection for integrated chromatographic separations in high‐throughput screening In: Gernaey KV, Huusom JK, Gani R, editors. 12th International Symposium on Process Systems Engineering and 25th European Symposium on Computer Aided Process Engineering. Computer Aided Chemical Engineering, Vol. 37 Amsterdam: Elsevier; 2015:2207–2212.

[39] Wiendahl M , Wierling PS , Nielsen J , Fomsgaard D , Krarup J , Staby A , Hubbuch J. High throughput screening for the design and optimization of chromatographic processes‐miniaturization, automation and parallelization of breakthrough and elution studies. Chem Eng Technol. 2008;31:893–903.

[40] Haimes YY , Lasdon LS , Wismer DA. On a bicriterion formulation of the problems of integrated system identification and system optimization. IEEE Trans Syst Man Cybern. 1971;1:296–297.

[41] Chankong V , Haimes YY. Multiobjective Decision Making: Theory and Methodology. New York: Elsevier Science; 1983.

[42] Ehrgott M , Ruzika S. Improved ε‐constraint method for multiobjective programming. J Optim Theory Appl. 2008;138:375–396.

[43] Mavrotas G. Effective implementation of the ε‐constraint method in multi‐objective mathematical programming problems. Appl Math Comput. 2009;213:455–465.

[44] Liu S , Papageorgiou LG. Multiobjective optimisation of production, distribution and capacity planning of global supply chains in the process industry. Omega 2013;41:369–382.

[45] Pareto V. Manuale di Economia Politica. Milan: Societa Editrice Libraria; 1906.

[46] Miettinen K. Nonlinear Multiobjective Optimization. Norwell: Kluwer Academic Publishers; 1999.

[47] Dinkelbach W. On nonlinear fractional programming. Manag Sci. 1967;13:492–498.

[48] Bradley JR , Arntzen BC. The simultaneous planning of production, capacity, and inventory in seasonal demand environments. Oper Res. 1999;47:795–806.

[49] Pochet Y , Warichet F. A tighter continuous time formulation for the cyclic scheduling of a mixed plant. Comput Chem Eng. 2008;32:2723–2744.

[50] You F , Castro PM , Grossmann IE. Dinkelbach's algorithm as an efficient method to solve a class of MINLP models for large‐scale cyclic scheduling problems. Comput Chem Eng. 2009;33:1879–1889.

[51] Billionnet A. Optimal selection of forest patches using integer and fractional programming. Oper Res Int J. 2010;10:1–26.

[52] Espinoza D , Fukasawa R , Goycoolea M. Lifting, tilting and fractional programming revisited. Oper Res Lett. 2010;38:559–563.

[53] Trinh K , Ferland J , Dinh T. A stochastic optimization method for solving the machine–part cell formation problem In: Huang DS, et al., editors. Avanced Intelligent Computing. Lecture Notes in Computer Science, Vol. 6838 Heidelberg: Springer‐Verlag; 2012:162–169.

[54] GAMS Development Corporation . GAMS: A User's Guide. Washington, DC: GAMS Development Corporation; 2014.

